# Leonurine Exerts Anti-Inflammatory Effects in Lipopolysaccharide (LPS)-Induced Endometritis by Modulating Mouse JAK-STAT/PI3K-Akt/PPAR Signaling Pathways

**DOI:** 10.3390/genes15070857

**Published:** 2024-06-29

**Authors:** Yongbin Shao, Yan Luo, Yaoqiang Sun, Jingbo Jiang, Zhiyuan Li, Zhen Wang, Mengmeng Wang, Xinli Gu

**Affiliations:** College of Animal Science and Technology, Shihezi University, Shihezi 832003, China; shaoyongbin@shzu.edu.cn (Y.S.); luoyan@shzu.edu.cn (Y.L.); sun586@shzu.edu.cn (Y.S.); j18841848296@163.com (J.J.); lizhiyuan@shzu.edu.cn (Z.L.); 19839912829@163.com (Z.W.); mw1879976@163.com (M.W.)

**Keywords:** anti-inflammatory, leonurine, LPS-induced endometritis, signaling pathway

## Abstract

Endometritis is a common disease in postpartum cows, characterized by delayed uterine recovery due to endometrial inflammation. Although antibiotics and hormones are commonly used, they have certain limitations. One potential alternative is using motherwort extract, specifically leonurine, which exhibits anti-inflammatory properties. However, leonurine’s exact molecular mechanism of action remains unclear. In this study, 40 mice were randomly divided into four groups: a control group, endometritis model group, LPS + leonurine group (30 mg/kg), and LPS + dexamethasone group (5 mg/kg). Transcriptomic analysis revealed that leonurine modulates multiple signaling pathways, including JAK-STAT/PI3K-Akt, and influences the expression of key genes, such as *Prlr*, *Socs2*, *Col1a1*, and *Akt1*. Furthermore, leonurine effectively reduces levels of inflammatory cytokines, such as tumor necrosis factor-alpha (TNF-α), interleukin (IL)-6, and IL-1β (*p* < 0.01), which play a crucial role in regulating acute endometritis. Additionally, leonurine helps maintain cholesterol homeostasis and attenuates inflammation through the peroxisome proliferator-activated receptor (PPAR) signaling pathway by modulating genes such as *Cyp27a1*, *Hmgcs1*, and *Scd2*. These findings suggest that leonurine has a protective effect against LPS-induced endometritis and that its anti-inflammatory properties involve multiple pathways and targets, which are potentially mediated by regulating signaling pathways such as JAK-STAT/PI3K-Akt and PPAR.

## 1. Introduction

Endometritis is the inflammation of the uterine lining, affecting several domestic animals such as cattle [1], horses [2], sheep [3], camels [4], dogs [5], and pigs [6]. Endometritis often occurs a few days after parturition, and requires prompt treatment, as the inflammation spreads easily, causing uterine plasma membrane or uterine periostitis, and often turns into chronic inflammation. Endometritis disrupts the estrous cycle, resulting in reproductive problems that lead to long-term infertility among livestock and cause economic losses to the dairy industry. Endometritis occurs in domestic animals due to various factors, but mainly from infections caused by pathogenic microbes, in particular, Gram-negative bacteria, such as *Escherichia coli* and *Fusobacterium necrophorum*. LPS is the outer membrane component of the cell wall of Gram-negative bacteria, and is widely used to study inflammation in different models, including acute lung injury, mastitis, and endometritis [7,8,9]. Currently, irrigation, antibiotics, and hormones are commonly used to treat endometritis; however, they have drawbacks, such as unsatisfactory therapeutic effects, drug resistance, and drug residues. Therefore, screening natural drug candidates from traditional Chinese medicine with few side effects for preventing and treating endometritis may help control endometritis among domestic animals.

Alkaloids are the main pharmacological component of motherwort, and the main active compound is leonurine, which has various pharmacological activities against cardiovascular, neurological, and uterine disorders, and is closely related to the traditional functions of motherwort [10,11]. Leonurine ameliorates chondrocyte cell and cartilage damage in mice by promoting autophagy and/or inhibiting inflammatory responses, and reduces interleukin 1β-induced inflammatory responses through the PI3K/AKT/NF-κB pathway [12]. Leonurine exerts nephroprotective effects against LPS-induced acute kidney injury in mice by inhibiting LPS-induced TLR4/NF-κB activation and pro-inflammatory cytokine production [13]. In rheumatoid arthritis fibroblast-like synoviocytes, treatment with leonurine decreases pro-inflammatory cytokines such as IL-1β, IL-6, IL-8, and TNF-α [14]. In mastitis treatment, leonurine exerts anti-inflammatory effects by inhibiting TLR4 expression, NF-κB activation, and p38, ERK, and JNK phosphorylation to upregulate IL-10 expression and downregulate TNF-α, IL-6, iNOS, and COX-2 expression [15]. Accordingly, to investigate the anti-inflammatory effects of leonurine on endometritis, this study used a uterine infusion of LPS to establish an animal model of endometritis, and explored its anti-inflammatory mechanisms using transcriptome sequencing technology to provide a theoretical basis for its mechanism of action and the clinical application of leonurine.

## 2. Materials and Methods

### 2.1. Laboratory Animals

The Biology Ethics Committee of Shihezi University approved this study (protocol number: A2023-82). Forty healthy female C57BL/6J mice, 6–8 weeks old and weighing 18–22 g, were obtained from the Zhengzhou Huiji Huaxing Laboratory Animal Center (Zhengzhou, China). Before the experiment, the mice were acclimatized to the experimental environment for 7 days (temperature of 24 ± 2 °C, relative humidity of 55% ± 5%, 12 h light and 12 h dark) with ad libitum access to food and water.

### 2.2. Grouping and Establishment of a Mouse Model of Endometritis

The 40 mice were randomly divided into four groups: the control group, endometritis model group (LPS), LPS + leonurine group (Leo, 30 mg/kg), and LPS + dexamethasone group (Dex, 5 mg/kg) (*n* = 10/group). The mice in the control group were fed a normal diet and injected with equal amounts of PBS as a placebo. The endometritis model was prepared by uterine perfusion with 50 µL of LPS (1 mg/mL) in mice in the LPS group. Mice in the Leo and Dex groups were injected with leonurine or dexamethasone intraperitoneally 1 h before modelling. They were then injected intraperitoneally with leonurine or dexamethasone every 6 h after modelling for a total of four administrations. Twenty-four hours after LPS infusion, the uterine tissues were collected from all the mice between 10 and 11 a.m. After flash freezing with liquid nitrogen, the uterine tissues were stored at −80 °C in a refrigerator. Transcriptomics sequencing analysis and cytokine detection were performed the day after sampling.

### 2.3. Cytokine Analyses

Cytokines such as TNF-α, IL-1β, and IL-6 were measured in uterine tissues collected randomly from 6 mice in each group. The cryopreserved uterine tissue was removed, weighed, and placed on an ice tray. PBS (pH 7.4) was added, and the samples were homogenized using a homogenizer. The supernatant was collected by centrifugation at 3000× *g* for 20 min. One portion was used for testing, and the rest was frozen. The procedure was performed following the manufacturer’s instructions for the ELISA kits (Biolegend, San Diego, CA, USA). The blank wells were used as a control and the absorbance (OD) of each well was measured sequentially at 450 nm wavelength using an ELISA reader to measure TNF-α, IL-1β, and IL-6 levels in the uterine tissue. The kits were validated in pre-tests, and the sensitivity of the ELISA kits, as well as the inter- and intra-variability of each ELISA kit, met the requirements.

### 2.4. Library Preparation for Transcriptome Sequencing

Uterine tissues from three mice were randomly selected from each group. The TRIzol reagent (Invitrogen, Carlsbad, CA, USA) was used to obtain total RNA from all samples following the manufacturer’s instructions. RNA integrity was assessed using the RNA Nano 6000 Assay Kit of the Bioanalyzer 2100 system (Agilent Technologies, Santa Clara, CA, USA). Total RNA was used as the input material for the RNA sample preparations.

In order to select cDNA fragments of, preferentially, 370~420 bp in length, the library fragments were purified with the AMPure XP system (Beckman Coulter, Beverly, CA, USA). Then, PCR was performed with Phusion High-Fidelity DNA polymerase, universal PCR primers, and Index (X) primer. At last, the PCR products were purified (AMPure XP system) and library quality was assessed on the Agilent Bioanalyzer 2100 system. The clustering of the index-coded samples was performed on a cBot Cluster Generation System using TruSeq PE Cluster Kit v3-cBot-HS (Illumia, San Diego, CA, USA), according to the manufacturer’s instructions.

After cluster generation, the library preparations were sequenced on an Illumina Novaseq platform and 150 bp paired-end reads were generated. Reference genome and gene model annotation files were downloaded from the genome website directly. The index of the reference genome was built using Hisat2 v2.0.5 and paired-end clean reads were aligned to the reference genome using Hisat2 v2.0.5. The mapped reads of each sample were assembled by StringTie (v1.3.3b) using a reference-based approach. FeatureCounts v1.5.0-p3 was used to count the read numbers mapped to each gene, and then the FPKM of each gene was calculated based on the length of the gene and the read counts mapped to this gene. Differential expression analyses between two comparison combinations were performed using the “DESeq2” R package (version 1.20.0). Differentially expressed genes (DEGs) were screened for |log2(FoldChange)| ≥ 1 and padj ≤ 0.05. The resulting *p*-values were adjusted using the Benjamini–Hochberg approach for controlling the false discovery rate. Genes with an adjusted *p*-value ≤ 0.05 found by DESeq2 were assigned as differentially expressed.

### 2.5. Gene Ontology (GO) and Kyoto Encyclopedia of Genes and Genomes (KEGG) Enrichment Analysis of DEGs

The GO enrichment analysis of the DEGs was implemented by the clusterProfiler (version 3.8.1) R package (https://www.bioconductor.org, accessed on 2 October 2023), in which gene length bias was corrected. GO terms with a corrected *p*-value less than 0.05 were considered significantly enriched by DEGs. The clusterProfiler (version 3.8.1) R package was used to perform the KEGG (http://www.genome.jp/kegg/, accessed on 2 October 2023) pathway enrichment analysis of the DEGs. Significantly enriched pathways were screened according to the hypergeometric test. The criterion for significant enrichment was set at *p* < 0.05. The Gene Set Enrichment Analysis (GSEA) tool (http://www.broadinstitute.org/gsea/index.jsp, accessed on 2 October 2023) was used to perform GSEA on the GO and KEGG datasets.

### 2.6. Real-Time Quantitative PCR (RT–qPCR) Analysis

Uterine tissues were randomly selected from three mice, each from the control, LPS, and Leo groups. Total RNA was extracted using RNA extraction solution (Servicebio, Wuhan, China) following the manufacturer’s instructions, and RNA concentration and purity were determined using Nanodrop 2000. Overconcentrated RNA was diluted at an appropriate ratio to give a final concentration of 100–500 ng/μL. The reaction mixture (20 μL) for reverse transcription comprised the following components: 4 μL of 5 × reaction buffer, 0.5 μL of Oligo (dT) 18 primer (100 μM), 0.5 μL of Random Hexamer primer (100 μM), 1 μL of Servicebio^®^RT Enzyme Mix, 10 μL of total RNA, and 4 μL of RNase-free water. The reverse transcription program settings were as follows: 25 °C for 5 min, 42 °C for 30 min, 85 °C for 5 s. The PCR reaction mixture comprised 7.5 μL of 2× qPCR mix, 1.5 μL of 2.5 μM forward and reverse primers (Table 1), 2.0 μL template cDNA, and 4.0 μL double-distilled water (RNase free). The PCR program (Takara Biotech, Dalian, China) settings were as follows: 95 °C for 30 s; 40 cycles each of 95 °C for 15 s and 60 °C for 30 s. Melting curves were obtained from 65 °C to 95 °C, with continuous fluorescence measurements obtained at every 0.5 °C increase in temperature. The expression of the PCR product was calculated based on the quantification of relative gene expression using ΔCt values, using GAPDH as the reference gene.

### 2.7. Statistical Analysis

ELISA Calc software (v 0.1)was used to analyze the ELISA results. Statistical significance was assessed by one-way analysis of variance (ANOVA), followed by Tukey’s comparison test using GraphPad Prism version 9 (GraphPad Software, San Diego, CA, USA). A value of *p* ≤ 0.05 was considered to indicate a statistically significant difference. All data were normality-checked and found to be normally distributed. No data from animals that met the inclusion/exclusion criteria appeared outside the above-mentioned test range.

## 3. Results

### 3.1. Effects of Leonurine on the Levels of Pro-Inflammatory Cytokines

**Figure 1:**
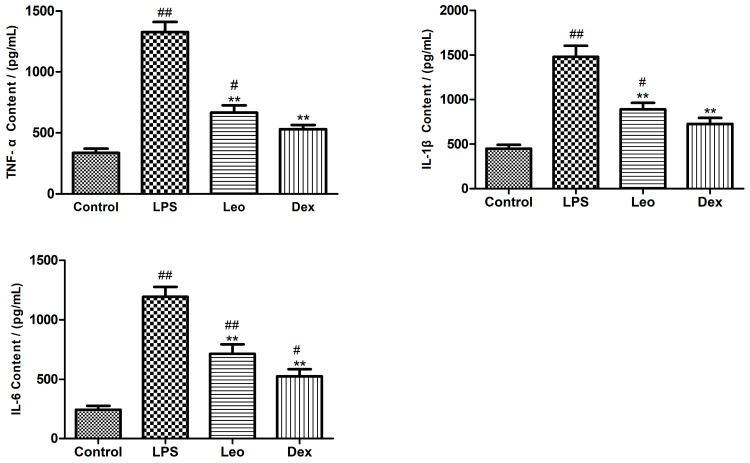
Effects of leonurine on cytokine production. The expressions of TNF-α, IL-6, and IL-1β were measured by ELISA. Data are represented as the mean ± standard error of the mean (SEM) from three independent experiments. # *p* < 0.05, ## *p* < 0.01 vs. control group; ** *p* < 0.01 vs. LPS group.

To evaluate the effects of leonurine on inflammatory cytokines in LPS-induced endometritis in mice, changes in TNF-α, IL-6, and IL-1β in uterine tissue homogenates were detected by ELISA. TNF-α, IL-6, and IL-1β levels (Figure 1) in the uterine tissues of mice in the LPS group were highly significantly increased compared with the control group (*p* < 0.01); however, TNF-α, IL-6, and IL-1β levels in the uterine tissues of mice in the Leo group and the Dex group were all highly significantly decreased compared with the LPS group (*p* < 0.01).

### 3.2. Differential Gene Analysis

**Figure 2:**
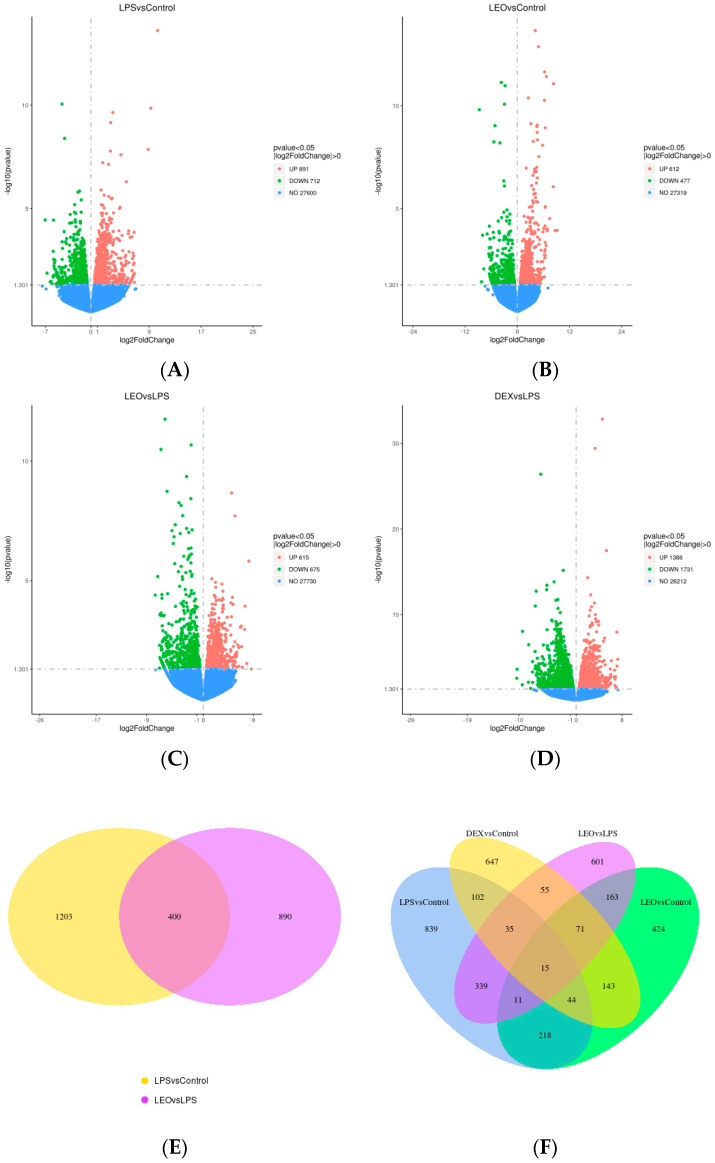

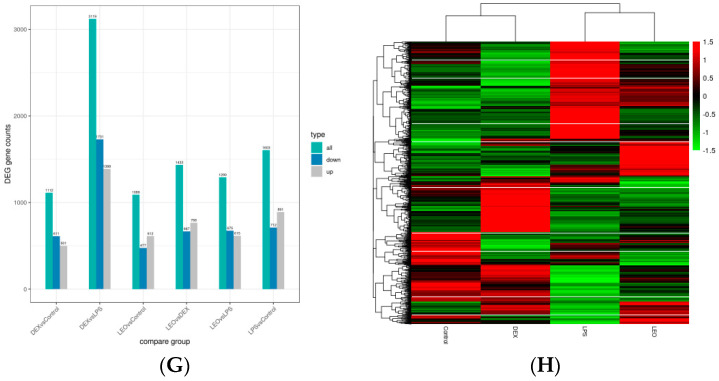
DEGs in various groups. (**A**) LPS vs. control. (**B**) Leo vs. control. (**C**) Leo vs. LPS. (**D**) Dex vs. LPS. (**E**) Venn diagram of the number of DEGs between LPS vs. control + Leo vs. LPS. (**F**) Venn diagram of the number of DEGs between LPS vs. control + LEO vs. control + Dex vs. control + Leo vs. LPS. (**G**) Statistical histogram of the number of DEGs in the combinations of the differential comparisons. (**H**) Heatmap for clustering of DEGs. Notes: The horizontal coordinates of the graphs indicate the fold change in gene expression in the two treatment and control groups. |log2(FoldChange)| and the vertical coordinates indicate the significance levels of the differences in gene expression in the two groups of treatment and control (−log10 padj or −log10 *p*-value). Upregulated genes are indicated by red dots, and downregulated genes are indicated by green dots.

To determine the molecular mechanism of leonurine in regulating endometritis and screen the pathways and target genes involved in the anti-inflammatory effects of leonurines, we analyzed the uterine tissues using transcriptomic technology and obtained DEGs from the different treatment groups (Figure 2). A total of 1603 DEGs were identified between the LPS and control groups, with 891 upregulated (red) and 712 downregulated DEGs (green) (Figure 2A). There were 1089 DEGs between the Leo and control groups, including 612 upregulated (red) and 477 downregulated DEGs (green) (Figure 2B). There were 1290 DEGs between the Leo and LPS groups, including 615 upregulated (red) and 675 downregulated (green) DEGs (Figure 2C). A total of 3119 DEGs were identified between the Dex and LPS groups, of which 1388 were upregulated (red) and 1731 were downregulated (green) (Figure 2D). The overlap of DEGs between different comparison combinations is shown in the Venn diagram, and the overlapping areas in Figure 2E,F indicate the number of DEGs shared between combinations. Figure 2G shows a histogram of the statistical number of differential genes in the differential comparison combinations. Figure 2H shows a heat map of differentially expressed gene clustering. These data suggest that the genes involved underwent significant changes after LPS stimulation and that using leonurine may block the tendencies of such gene changes to a certain extent.

### 3.3. GO Enrichment Analysis of the DEGs

**Figure 3:**
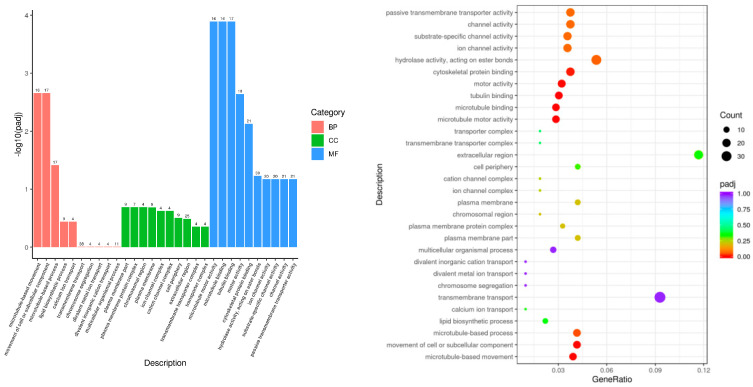
Histogram and scatterplot of GO enrichment analysis of LPS vs. control.

The DEG sets were mapped to each term in the GO database to analyze the number of genes that were mapped to the biological process (BP), cellular component (CC), and molecular function (MF) terms. The GO annotation results indicated that 675 GO terms were enriched in the DEGs between the LPS and control groups. These terms included 362 BP terms, 72 CC terms, and 241 MF terms. The top 30 enriched terms (Figure 3) included microtubule-based movement, movement of cell or subcellular component, microtubule-based process, microtubule motor activity, microtubule binding, tubulin binding, motor activity, and cytoskeletal protein binding. This result suggested that, after LPS-induced endometritis in mice, inflammation had a greater effect on the gene expression of kinesin superfamily proteins (Kifs), suggesting that LPS-induced endometritis has a greater effect on the function and morphology of cells in uterine tissue.

**Figure 4:**
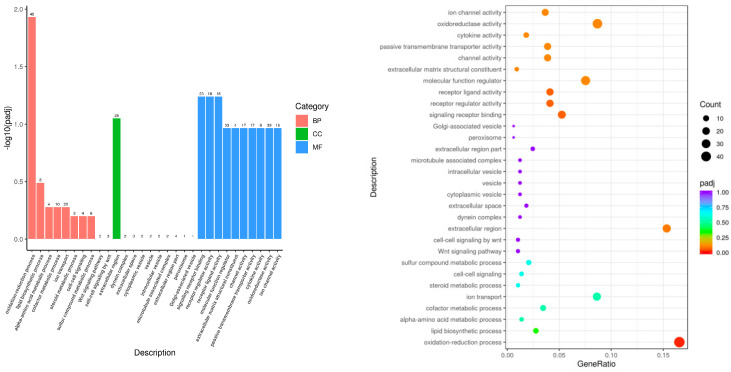
Histogram and scatterplot of GO enrichment analysis of Leo vs. LPS.

The GO functional enrichment analysis showed that the DEGs between the Leo and LPS groups were mainly mapped to the BP (such as the oxidation–reduction process, the Wnt signaling pathway, and cell–cell signaling by wnt), MF (such as signaling receptor binding, receptor regulator activity, and cytokine activity), and CC areas (such as the extracellular region, extracellular space, and microtubule-associated complex) (Figure 4). The GO enrichment analysis revealed that leonurine might alter the expression of genes associated with lipid synthesis, energy metabolism, inflammatory pathways, and immune responses in mice with endometritis.

### 3.4. KEGG Enrichment Analysis of the DEGs

**Figure 5:**
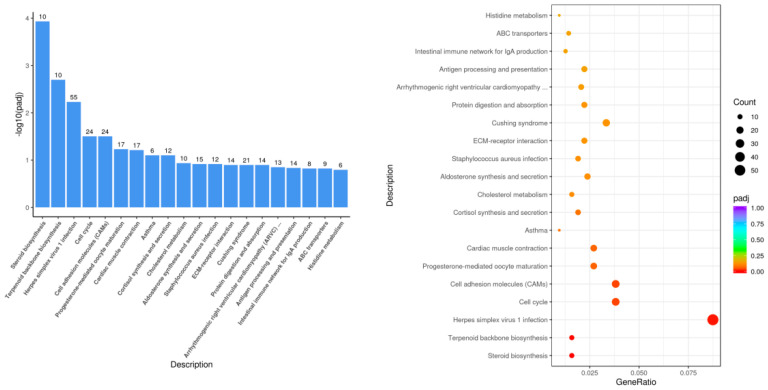
Histogram and scatterplot of KEGG enrichment analysis of LPS vs. control.

The KEGG pathways with the most significant differential gene enrichment in the LPS group compared with the control group (Figure 5) were steroid biosynthesis, terpenoid backbone biosynthesis, cell cycle, cell adhesion molecules, and other biological pathways.

**Figure 6:**
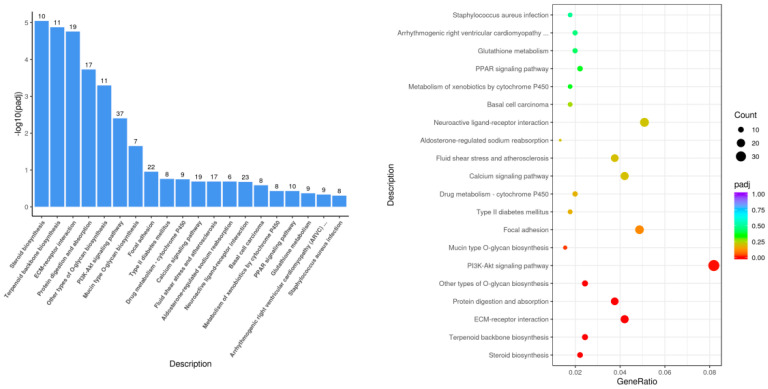
Histogram and scatterplot of KEGG enrichment analysis of Leo vs. LPS.

The KEGG pathway enrichment results showed that 295 pathways were enriched in the Leo group compared with those in the model group (Figure 6). The KEGG pathways with the most significant differential gene enrichment were steroid biosynthesis, protein digestion and absorption, the PI3K-Akt signaling pathway, the PPAR signaling pathway, and other biological pathways. There was also significant enrichment in inflammatory signaling pathways, including the Wnt signaling pathway and the MAPK signaling pathways. Genes with significant differences in the above-mentioned pathways included *Hmgcr*, *Mapk10*, *Gadd45g*, *Wnt5b*, *Igf1*, *Ccl17*, *Cyp27a1*, *Prlr*, *Socs2*, *Hmgcs1*, *Scd2*, *Creb3l3*, *Prkcq*, *Ror2*, *Il11*, *Casp1*, *Col1a1*, *Akt1*, and several other genes.

### 3.5. RT-qPCR Verified the Expression of Significantly Different Genes

**Figure 7:**
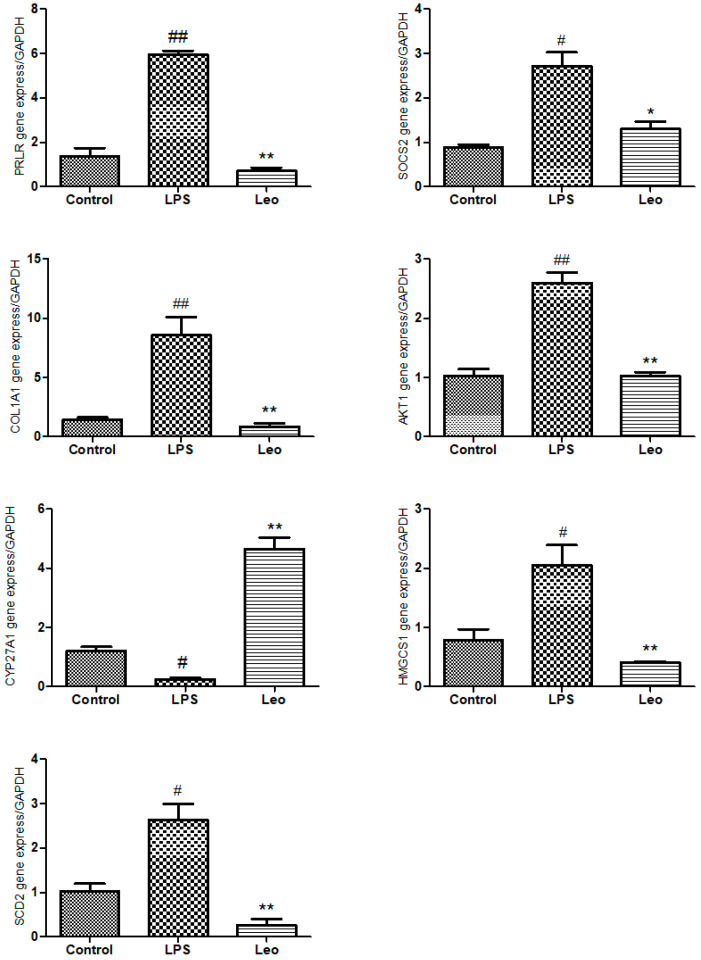
Effect of leonurine on differential gene expression. The expressions of *Prlr*, *Socs2*, *Col1a1*, *Akt1*, *Cyp27a1*, *Hmgcs1*, and *Scd2* genes were measured by RT-qPCR method. # *p* < 0.05 vs. control group; ## *p* < 0.01 vs. control group; * *p* < 0.05, ** *p* < 0.01 vs. LPS group.

Compared with the control group (Figure 7), the relative expression of *Cyp27a1* (*p* < 0.05) was downregulated, and the relative expressions of *Socs2* (*p* < 0.05), *Hmgcs1* (*p* < 0.05), *Scd2* (*p* < 0.05), *Prlr* (*p* < 0.01), *Col1a1* (*p* < 0.01), and *Akt1* (*p* < 0.01) were upregulated in the LPS group. Compared with the LPS group, the Leo group showed a significantly upregulated relative expression of *Cyp27a1* (*p* < 0.01) and significantly downregulated relative expressions of *Socs2* (*p* < 0.05), *Prlr* (*p* < 0.01), *Hmgcs1* (*p* < 0.01), *Scd2* (*p* < 0.01), *Col1a1* (*p* < 0.01), and *Akt1* (*p* < 0.01). The relative expression trends of these seven genes were consistent with the results of the transcriptomic analysis and RT-qPCR validation.

## 4. Discussion

Endometritis is defined as inflammation of the endometrium in the absence of systemic signs, and is associated with delayed uterine involution. This condition often leads to reproductive problems in dairy cows. Although antibiotics are widely used to treat endometritis in dairy cows, long-term treatment with high doses of antibiotics can lead to bacterial resistance and drug residues, posing unavoidable problems. Therefore, alternative pharmacological strategies for endometritis treatment warrant exploration from other perspectives. The research in this study demonstrated that the transvaginal instillation of a specific amount of LPS induced acute endometritis in mice, leading to increased levels of pro-inflammatory cytokines such as TNF-α, IL-6, and IL-1β. However, leonurine effectively inhibited the levels of these inflammatory factors during the acute phase of endometritis. Notably, the findings indicated that there were 1603 DEGs identified between the LPS and control groups, including 891 upregulated and 712 downregulated genes. The following genes were regulated by LPS through the JAK-STAT/PI3K-Akt pathways: *Angpt4*, *Thbs4*, *Prlr*, *Tek*, *Eif4ebp1*, *Ddit4*, *Rps6kb2*, *Epha2*, *Creb3l3*, *Tnc*, *Tcl1b1*, *Col1a1*, *Il4*, *Itga9*, *Map2k2*, *Lpar6*, *Gm29811*, *Akt1*, *Fgf10*, *Pck1*, *Il4ra*, *Col6a5*, *Fgfr2*, *Itgb8*, *Gnb2*, *Lpar4*, *Lamb3*, *Itga11*, *Socs2*, *Lepr*, *Il12a*, and *Il11*. Importantly, leonurine regulated LPS-induced endometritis through the modulation of several signaling pathways, including WNT, MAPK, AMPK, and JAK-STAT/PI3K-AKT, with JAK-STAT/PI3K-AKT being one of the essential pathways. The following genes were regulated by leonurine through the JAK-STAT/PI3K-Akt pathways: *Itgb3*, *Col4a4*, *Ngfr*, *Angpt4*, *Creb3l3*, *Tgfa*, *Igf1r*, *Igf1*, *Irs1*, *Areg*, *Sgk1*, *Col1a1*, *Kit*, *Efna3*, *Angpt1*, *Bdnf*, *Fgfr3*, *Fgf16*, *Ntrk1*, *Kdr*, *Flt3l*, *Ppp2r2c*, *Rptoros*, *Hsp90b1*, *Prlr*, *Socs2*, *Socs1*, *Il12rb2*, *Il11*, and *Akt1*. Furthermore, leonurine was observed to modulate overall cholesterol stability and energy metabolism in macrophages by influencing the expression of genes like *Cyp27a1*, *Hmgcs1*, and *Scd2*. By regulating multiple pathways and targets, leonurine prevents LPS-induced acute inflammation, promotes recovery, and shows potential for regulating acute endometritis.

In the functional classification of inflammatory factors, TNF-α, IL-6, and IL-1β are considered key pro-inflammatory factors regulating innate immunity, synergistically mediating the acute-phase response to inflammation. They play pivotal roles in the inflammatory cascade and are important indicators of disease progression and inflammatory status. The measurement of TNF-α, IL-6, and IL-1β levels can provide insights into the severity of inflammatory conditions. In this study, the results showed that a uterine infusion of 50 μL LPS (1 mg/mL) significantly increased TNF-α, IL-6, and IL-1β levels in the uterine tissue of mice, whereas 30 mg/kg leonurine significantly reduced their levels in LPS-induced mouse uterine tissue, suggesting that leonurine may exert anti-inflammatory effects on LPS-induced endometritis by inhibiting proinflammatory cytokine release.

The JAK/STAT pathway is a prominent signaling cascade activated by several critical cytokines implicated in sepsis, such as IFN-γ, IL-4, IL-6, IL-10, and IL-12 [16]. *Prlr* belongs to the cytokine type I receptor superfamily, and similar to all cytokine receptors, it is a single-pass transmembrane chain. *Prlr* expression can be regulated by various stimuli, such as inflammatory mediators. Prlrs also activate a subset of STAT proteins, including STAT1, STAT3, STAT5A, and STAT5B [17]. In various infection models like Toxoplasma gondii, Trypanosoma cruzi, and Plasmodium berghei Anka, *Socs2* has been recognized as a modulator of innate and adaptive immune responses. In these models, the involvement of *Socs2* is complex and involves Th1, Th2, Th17, and T-regulatory cell generation and differentiation [18,19,20,21]. *Socs2* contributes to the induction of neuroinflammation in the early and peak phases of experimental autoimmune encephalomyelitis [22]. The current study observed that endometrial inflammation significantly increased the expression of the *Prlr* gene (*p* < 0.01) and *Socs2* (*p* < 0.05) in the uterine tissues of mice after LPS treatment. Treatment with leonurine attenuated the inflammation-induced increases in *Prlr* and *Socs2* expression. This suggests that leonurine may inhibit the JAK/STAT pathway during acute inflammation by regulating the relative expression of *Prlr* and *Socs2* genes to play an anti-inflammatory role. This modulation could prevent the exacerbation of acute inflammation, mitigate uterine tissue damage, and facilitate recovery from endometritis.

The PI3K/AKT signaling pathway plays a crucial role in cell survival, promotes cell proliferation, and inhibits apoptosis. It also regulates inflammation and immune responses. *Akt1* regulates the innate immunity in macrophages by mediating mitochondrial H_2_O_2_ production [23]. Collagen type I alpha 1 chain (*Col1a1*) is a biomarker for collagen metabolism and tissue fibrosis [24]. *MiR-92b* has been implicated in protecting against LPS-induced inflammatory injury by inhibiting the TLR 4 signaling of the PTEN/PI 3 K/AKT/β-catenin axis [25]. Interferon-τ may protect against inflammatory injury by inhibiting TLR4/NF-κB activation through the regulation of the PI3K/AKT/β-catenin/FoxO1 axis [26]. Endometrial inflammation during LPS-induced acute endometritis significantly upregulated *Col1a1* and *Akt1* gene expression in mouse uterine tissues (*p* < 0.01), whereas leonurine was able to significantly reduce the inflammation-induced increase in *Col1a1* and *Akt1* gene expression. This suggests that leonurine may alleviate fibrosis caused by LPS-induced acute endometrial inflammation by regulating the innate immunity of macrophages and the expression of key genes in the signaling pathway, inhibiting the PI3K/AKT signaling pathway, and alleviating inflammation-induced fibrosis in the uterine tissue. Thus, leonurine may exert protective effects against acute inflammatory injury in LPS-induced endometritis.

The PPAR family plays a critical role in regulating energy balance and metabolic functions [27]. Cytochrome P450 family 27 subfamily A member 1 (*Cyp27a1*) is a rate-limiting enzyme in the bile acid synthesis bypass pathway. It is important for cholesterol homeostasis and is closely associated with tumorigenesis. Baicalin increases the expression of bile acid synthase (*Cyp27a1*) and significantly inhibits hepatic inflammatory responses in rats with EIC [28]. 3-Hydroxy-3-methylglutaryl coenzyme A synthase 1 (*Hmgcs1*) is the rate-limiting enzyme in the mevalonate (MVA) pathway, catalyzing the synthesis of HMG-CoA, and is a key enzyme in the cholesterol synthesis pathway. In human cancer cells, PPAR-α, a key transcriptional regulator of lipid metabolism, cross-talks with SREBP to regulate the expression of *Hmgcs1* and *Msmo1* [29]. Stearoyl-coenzyme A desaturase-2 (*Scd2*) is a member of the SCD family of enzymes, and acts as both an energy source and a signaling molecule [30]. LPS induces SCD expression, resulting in the formation of foam cells, which are macrophages that contain high levels of fatty acids. Therefore, SCD may play a crucial role in regulating energy metabolism [31]. The present study observed that inflammation during LPS-induced acute endometritis disrupts cellular processes such as nutrient uptake and metabolic regulation. Additionally, leonurine was found to enhance bile acid metabolism by regulating cholesterol synthesis and transport in macrophages, thereby favorably influencing overall cholesterol homeostasis. However, further research is needed to elucidate the precise mechanisms by which leonurine regulates energy and cholesterol metabolisms.

## 5. Conclusions

In conclusion, the present study demonstrated that leonurine exhibits potent anti-inflammatory properties. The mechanisms underlying leonurine’s anti-inflammatory effects involve several molecular pathways. It inhibits the expression of *Prlr*, *Socs2*, *Col1a1*, and *Akt1* genes within the JAK-STAT/PI3K-Akt pathway, and downregulates the pro-inflammatory cytokines TNF-α, IL-6, and IL-1β. Additionally, leonurine modulates the expression of *Cyp27a1*, *Hmgcs1*, and *Scd2* in the PPAR pathway, thereby improving cholesterol and energy metabolism and maintaining overall cholesterol homeostasis. In summary, leonurine acts through multiple pathways and regulates gene expression to exert its anti-inflammatory effects, as demonstrated in its ability to mitigate LPS-induced endometritis in mice. These findings suggest that leonurine holds promise as a potential therapeutic agent for the treatment of endometritis.

## Figures and Tables

**Table 1:** List of primers used in this study.

Genes	Forward Primer	Reverse Primer
*GAPDH*	GAAGGTGGTGAAGCAGGCATCT	CGGCATCGAAGGTGGAAGAGTG
*Prlr*	ACTCACTCCTCTCCTGCGTTCT	TGCGATGCTCACCTCCACAGA
*Socs2*	CCACCTCGCCACATTCCATCTT	GCCGTCAATCATCTCAGCAAGC
*Col1a1*	GCTCGTGGATTGCCTGGAACA	CAGCACCAACAGCACCATCGT
*Akt1*	TGGACTTCCGATCAGGCTCACC	TGGCGACGATGACCTCCTTCTT
*Cyp27a1*	AAGGACCACCGAGACCACAAGG	GTGATGGCTTCCAAGGCAAGGT
*Hmgcs1*	TGGTTCCCTGGCTTCTGTCCTG	TCCTGGTGTGGCGTCTTGTGT
*Scd2*	TCCTGCTGATGTGCTTCGTCCT	AGGCGTGGTGGTAGTTGTGGAA

## Data Availability

The original contributions presented in the study are included in the article/Supplementary Materials, further inquiries can be directed to the corresponding author.

## Supplementary Materials

The following supporting information can be downloaded at: https://www.mdpi.com/article/10.3390/genes15070857/s1, Table S1: Differentially expressed genes between the Leo group and the LPS group; Table S2: Differentially expressed genes between the LPS group and the control group; Table S3: GO annotation of differentially expressed genes between the Leo group and the LPS group; Table S4: GO annotation of differentially expressed genes between the LPS group and the control group; Table S5: KEGG enrichment analysis of differentially expressed genes between the Leo group and the LPS group; Table S6: KEGG enrichment analysis of differentially expressed genes between the LPS group and the control group.
